# Expert Consensus on the Use of Lurasidone for Bipolar I Depression in Asian Patients

**DOI:** 10.31083/AP49358

**Published:** 2026-06-30

**Authors:** Ahmad Hatim Sulaiman, Mo-Ching Eileena Chui, Ronnachai Kongsakon, Jen-Chin Lee, Chien-Heng Lin, Beng Yeong Ng, Marcus Wee Lun Tan, Joe Hang Ting, Meor Hakimi Meor Idris

**Affiliations:** ^1^Department of Psychological Medicine, University Malaya, 50603 Kuala Lumpur, Malaysia; ^2^Department of Psychiatry, Queen Mary Hospital and KS Faculty of Medicine, University of Hong Kong, Hong Kong, China; ^3^Department of Psychiatry, Mahidol University, 10400 Bangkok, Thailand; ^4^Department of General Psychiatry, Taoyuan Psychiatric Center, 330053 Taoyuan, Taiwan; ^5^National Taiwan University Hospital, Hsin-Chu Branch, Hsinchu County, Taiwan; ^6^Graduate Institute of Clinical Medicine, College of Medicine, National Taiwan University, 302645 Taipei, Taiwan; ^7^Ng Beng Yeong Psych Medicine Clinic, 329565 Singapore, Singapore; ^8^Garden Grove Clinic, 427646 Singapore, Singapore; ^9^Ting Specialist Clinic, 55100 Kuala Lumpur, Malaysia; ^10^Marubeni Pharmaceuticals Malaysia, 50470 Kuala Lumpur, Malaysia

**Keywords:** bipolar disorder, consensus statement, lurasidone, antipsychotic agents, Southeast Asian people, East Asian people

## Abstract

​​Eight psychiatrists from Malaysia, Singapore, Taiwan, Hong Kong, and Thailand participated in three meetings using the RAND Corporation/University of California Los Angeles (RAND/UCLA) appropriateness method to establish expert consensus on the use of lurasidone for bipolar I depression in Asian patients. The first meeting generated clinical questions and statements for voting, the second refined these statements, and the third addressed disagreements or areas of uncertainty. Consensus was defined as ≥66% agreement that a statement was appropriate or extremely appropriate. Nearly all proposed statements achieved consensus, including the use of lurasidone as a first-line treatment for first-episode bipolar I depression in adults, with a starting dose of 20 mg/day and titration up to 120 mg/day as needed, as well as its use as adjunctive therapy with mood stabilizers. For elderly patients, a lower dose range of 20–80 mg/day was recommended, while for children and adolescents, a dose range of 20–80 mg/day with careful monitoring was suggested. Lurasidone was considered to have a lower risk of inducing mania or hypomania compared with placebo, conventional antidepressants, and certain antipsychotics, and was associated with a favorable cardiometabolic profile and generally manageable side effects, including nausea, akathisia, and somnolence. Caution was advised when prescribing lurasidone during pregnancy, particularly in the first trimester. This expert consensus provides practical guidance for the use of lurasidone in the treatment of bipolar I depression in Asian patients, complementing existing treatment guidelines and addressing dosing strategies, side effect management, and use in special populations.

## Main Points

1. This RAND Corporation/University of California Los Angeles (RAND/UCLA) Expert Consensus among 8 Asian psychiatrists provided practical dosing/switching guidance for treating Asian patients with bipolar I depression.

2. Lurasidone is recommended as a first-line treatment for first-episode bipolar I depression in Asian adults, starting at 20 mg/day and titrating to 60–80 mg/day (up to 120 mg/day if necessary).

3. Lower doses (20–80 mg/day) should be used with caution in elderly and child/adolescent patients.

4. Lurasidone is appropriate as adjunctive therapy with mood stabilizers (e.g., lithium, valproate) when monotherapy response is inadequate or for maintenance.

5. Lurasidone has a favorable cardiometabolic profile, minimal weight gain, manageable side effects (akathisia, nausea, somnolence), and a low risk of mania switching.

## 1. Introduction

Bipolar disorder is a chronic, recurrent psychiatric condition affecting approximately 2.5% of individuals globally [1] and is characterized by diminished function, cognitive impairment, and reduced quality of life [2]. Recent studies using broadened diagnostic criteria have suggested higher lifetime prevalence rates (4.5%–10.9%) and a greater prevalence of bipolar I disorder (BD-I) in Asians than in Caucasians. A cross-sectional survey from Singapore [3] between 2016 and 2018 reported a 1.5% lifetime weighted prevalence of BD-I and increased rates of severe mania/hypomania symptoms and depression. According to a recent meta-analysis [4], more than half of people in Asia with bipolar disorder had a manic predominance (weighted prevalence = 52.7%), whereas depressive predominance occurred in around one-fifth of patients (weighted prevalence = 20.5%). Bipolar disorder typically manifests between the ages of 15 and 19. Earlier onset is associated with poorer outcomes [5], including substance abuse, higher mortality [6], and psychiatric comorbidities [7]. Affected adolescents reported a lower quality of life than did non-BD-I peers [8]. Elderly patients (≥60 years) [9] comprise 25% of the bipolar population. This group has a 1% BD-I diagnosis rate, and 70% of those are female [10]. However, the lifespan incidence of BD-I appears comparable between sexes [11], but the risk of antenatal and postpartum bipolar recurrence significantly increases in females [11,12], and can have negative effects on both the mother and neonate.

BD management must balance symptom management and treatment risks [13,14] and includes the use of pharmacotherapy [15] with drugs such as antipsychotics to regulate manic or hypomanic episodes. Selection of antipsychotics requires evaluation of efficacy for the BD severity level, side effect tolerability, and treatment accessibility. Antipsychotics are available in typical and atypical forms, with atypical antipsychotics being better at managing negative symptoms, cognitive impairment, relapse, extrapyramidal symptoms, and hyperprolactinemia.

Lurasidone hydrochloride (“lurasidone”), a second-generation antipsychotic (SGA), was indicated for schizophrenia and bipolar I depression in Asia at the study’s inception. It has been approved for bipolar depression in several Asian countries [16,17] for several years. Lurasidone exhibits both antipsychotic and antidepressant actions [18], has antagonist activity at dopamine D2 and serotonin 5-hydroxytryptamine receptor 2A (5-HT2A) receptors, high affinity for serotonin 5-HT7 receptors (antagonism), and is a partial 5-HT1A receptor agonist. With minimal effects on body weight and low risks for significant changes in glucose, lipids, or electrocardiogram parameters [18,19], its side effect profile is more tolerable [20] than that of other antipsychotics.

The Program to Evaluate the Antidepressant Impact of Lurasidone (PREVAIL) comprised three 6-week, randomized, double-blind, placebo-controlled trials on the effectiveness of lurasidone in bipolar depression. In PREVAIL-1 [21], lurasidone (20–120 mg/day) as adjunctive therapy to lithium or valproate reduced depression severity and anxiety symptoms, and improved quality of life and daily functioning. The incidence of treatment-emergent mania (TEM) was similar to that of placebo, although somnolence, akathisia, and extrapyramidal events were slightly more frequent. In PREVAIL-2 [22], lurasidone monotherapy reduced depression severity more than did placebo, with increasing improvements from week 2, and also significantly improved anxiety symptoms, quality of life, and daily function. In PREVAIL-2, TEM occurred in 3.7% of patients on lurasidone 20–60 mg/day, 1.9% on lurasidone 80–120 mg/day, and 1.9% on placebo. Nausea affected 10.4%, 17.4%, and 7.7%, respectively; akathisia 7.9%, 10.8%, and 2.4%, respectively; and extrapyramidal events, sedation, and vomiting showed similar dose-dependent patterns to placebo. PREVAIL-3 [23] assessed lurasidone as adjunctive therapy to lithium or valproate among patients receiving mood stabilizers retrospectively or prospectively. Depression severity did not differ significantly between lurasidone and placebo from baseline to week 6. However, a trend favoring lurasidone emerged between weeks 2–5. In pre-planned analyses, patients treated retrospectively with mood stabilizers had significantly greater improvements in the Montgomery–Åsberg Depression Rating Scale (MADRS) score than did those treated prospectively. Lurasidone led to slightly more frequent akathisia than did placebo (14.1% vs 5.3%), as well as somnolence (11.9% vs 4.7%) and extrapyramidal events (12.4% vs 7.6%). The PREVAIL-1 trial reported that the most common adverse events with lurasidone were nausea, headache, akathisia, and somnolence, with minimal changes in weight, lipids, and metabolic parameters [21]. All PREVAIL trials [21,22,23,24] reported few changes in weight, lipid levels, and glycemic control with lurasidone.

Those studies demonstrated lurasidone efficacy, but treatment guidelines for its use in BD-I varied, with no consensus on dosage in bipolar depression [25,26]. Recommendations suggested a wide dose range (e.g., 20–120 mg) [27,28], indicating uncertainty about its optimal use. The Canadian Network for Mood and Anxiety Treatments and the International Society for Bipolar Disorders [26] guidelines recommended lurasidone monotherapy or adjunctive lithium/valproate therapy, but not first-line, adjunctive lamotrigine [29]. First-line lurasidone was recommended by the British Association for Psychopharmacology [30] for acute bipolar depression and by the Royal Australian and New Zealand College of Psychiatrists [31] for bipolar depression. Guidelines also varied in Asia. In Malaysia [32], lurasidone was recommended as monotherapy or in combination with a mood stabilizer for acute depressive episodes (starting with 20 mg once daily and titrating in 20 mg increments at longer than 2-day intervals, up to 120 mg once daily), and for children/adolescents with BD depressive episodes (20–40 mg once daily, up to 80 mg/day). A recent Taiwanese consensus [27] recommended lurasidone monotherapy for the acute depressive phase, followed by adjunctive lurasidone and quetiapine monotherapy and mixed features/episodes in the acute and maintenance phases. However, expert recommendations or consensus on lurasidone for bipolar I depression have not been published for Asian patients by psychiatrists practicing in Asia. To achieve the best possible care for our patients, we convened consensus meetings to discuss, propose, and vote on statements for the use of lurasidone.

## 2. Methods

### 2.1 Expert Panel Selection and Composition

Excluding one author (MH was present only as an industry-affiliated observer and to provide relevant information on lurasidone), eight psychiatrists were selected as expert panelists. The criteria used to select them were: (1) documented clinical expertise in treating patients with BD-I; (2) geographic representation limited to five Asian regions/territories where lurasidone is currently approved and marketed by Marubeni Pharmaceuticals Asia Pacific; and (3) inclusion of clinical subspecialties relevant to bipolar disorder treatment across the lifespan.

The final panel composition was as follows: Malaysia (*n *= 2), Singapore (*n *= 2), Taiwan (*n *= 2); Hong Kong (*n *= 1); and Thailand (*n *= 1). Seven panelists were trained in general adult psychiatry, and one panelist (CHL) specialized in child-adolescent psychiatry, providing representation across age groups and maintaining focus on the most clinically prevalent population (adults with BD-I).

Eight experts were considered sufficient for three reasons: the RAND Corporation/University of California Los Angeles (RAND/UCLA) method is validated for panels of 7–13 members [33]; this size aligned with comparable regional initiatives [27,32]; and restricting inclusion to countries with regulatory approval of lurasidone provided natural boundaries for panel selection. Although the panel did not represent all Asian regions or psychiatry subspecialties (e.g., geriatric or perinatal psychiatry), its composition balanced practical feasibility with methodological rigor and clinical relevance.

### 2.2 Development of Clinical Questions and Statements

The first author (AH) conducted an initial review of the lurasidone literature and identified clinically relevant gaps in Asia-Pacific treatment guidance, which were used to formulate the draft clinical questions. These questions were collectively refined by all eight panelists during the first structured advisory board meeting (May 2024) and finalized through full panel consensus at a second meeting (October 2024). During this phase, the industry-affiliated observer (MH) reviewed the draft questions and supporting literature and provided evidence-based feedback on their clinical relevance and alignment with the current pharmacological evidence base for lurasidone. The observer’s role was explicitly limited to literature support and question refinement; the observer did not participate in voting, consensus decisions, or interpretation of voting results. The final advisory board meeting (February 2025) was devoted exclusively to voting on the appropriateness of recommendation statements and consensus determination.

### 2.3 Consensus Process

The RAND/UCLA appropriateness method was used to establish a consensus on the use of lurasidone for bipolar I depression. Briefly, the voters participated in three meetings: one to develop a list of key clinical questions and potential recommendation statements for a consensus vote, one to review and refine the questions and recommendation statements for the consensus vote, and one to discuss areas of disagreement and uncertain ratings resulting from the consensus vote.

### 2.4 Consensus Rating of Recommendation Statements

Questions and recommendations were distributed electronically via Microsoft Forms, which allowed the voters to independently rate each recommendation statement on a 5-point Likert scale for appropriateness (1 = *extremely inappropriate*; 2 = *inappropriate*; 3 = *uncertain*; 4 = *appropriate*; and 5 = *extremely appropriate*). Their responses were then consolidated, graphically represented, and analyzed to determine a level of agreement for each question and recommendation statement.

### 2.5 Definition of Consensus and Agreement Levels

Consensus was defined a priori as ≥66% (≥6/8) panelists rating statements as *appropriate* or *extremely appropriate* (Likert scores). Statements below this threshold were excluded from final recommendations. Statements achieving consensus were classified post hoc by agreement intensity: Moderate Consensus: 66%–79% agreement; High Consensus: 80%–94% agreement, and Full Consensus: ≥95% agreement. All final recommendations met the ≥66% threshold. Full consensus statements represented the strongest endorsement; moderate consensus indicated some expert disagreement.

## 3. Results

The voters had an average of 24 years in psychiatric practice (range: 15–30 years), 19 years of treating bipolar depression in Asian patients (range: 10–30 years), 5 years of experience in prescribing lurasidone for all approved indications (range: 2–8 years), and 4 years of experience in prescribing lurasidone for bipolar depression in Asian patients (range: 2–6 years).

Overall, due to the pre-voting review and refinement process, all statements achieved agreement and therefore consensus (see **Supplementary Table 1** for summary). Statements highlighted in this section were those with one or more votes for *uncertain, inappropriate, *or *extremely inappropriate.*

**Fig. 1.**
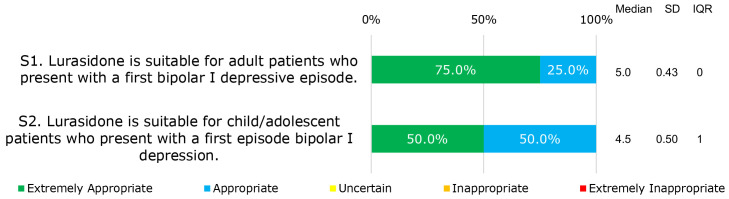
**Question 1: Lurasidone for patients who present with first-episode bipolar I depression**. SD, standard deviation; IQR, interquartile range; S, statement.

**Fig. 2.**
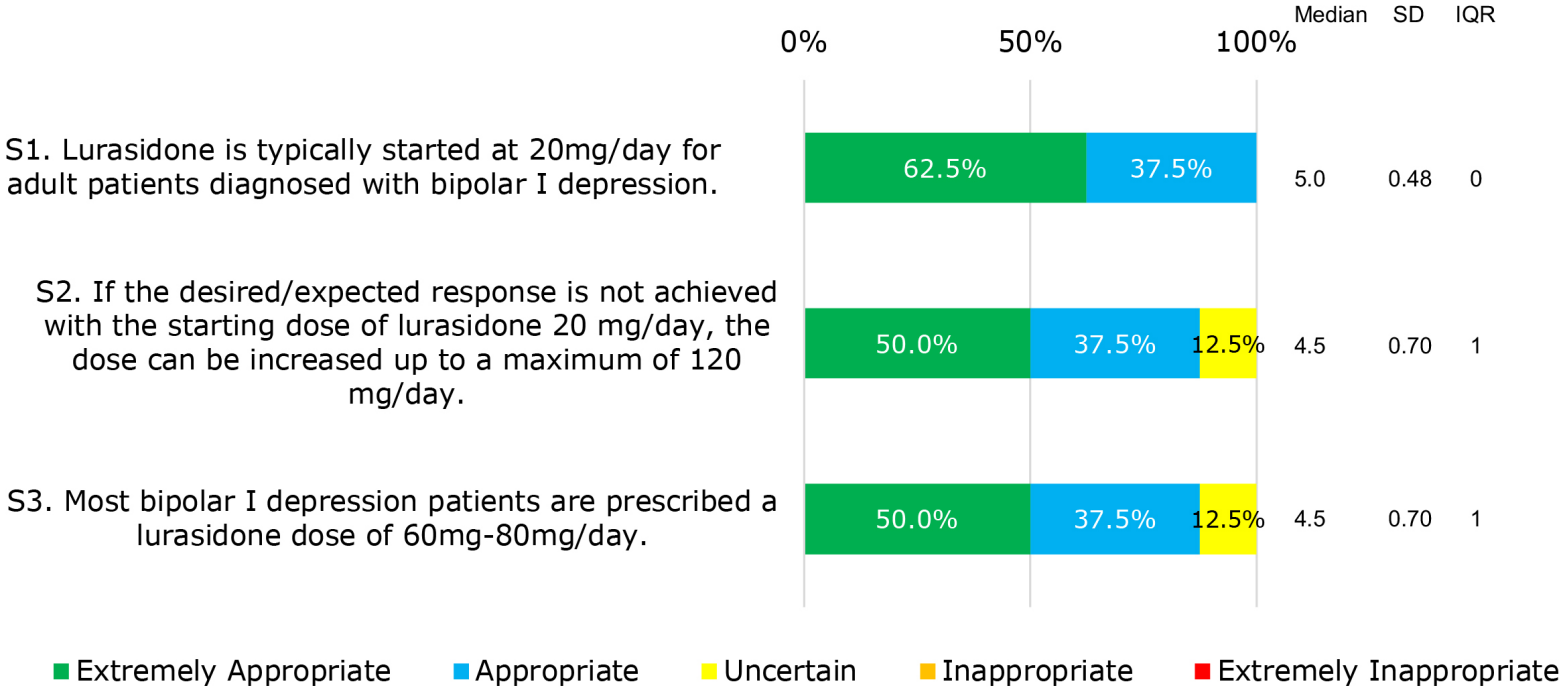
**Question 2: Lurasidone usage in adult patients with bipolar I depression**.

The voters agreed on all statements regarding lurasidone usage in patients who present with first-episode bipolar I depression (Fig. 1). For lurasidone usage in adult patients with BD-I [Fig. 2; Question 2 (Q2)], one voter (12.5%) was *uncertain* about increasing the lurasidone dose up to 120 mg/day if the starting dose (20 mg/day) did not achieve the desired/expected response [Statement 2 (S2)], and one voter was *uncertain *that most patients were prescribed 60–80 mg/day of lurasidone [Statement 3 (S3)].

**Fig. 3.**
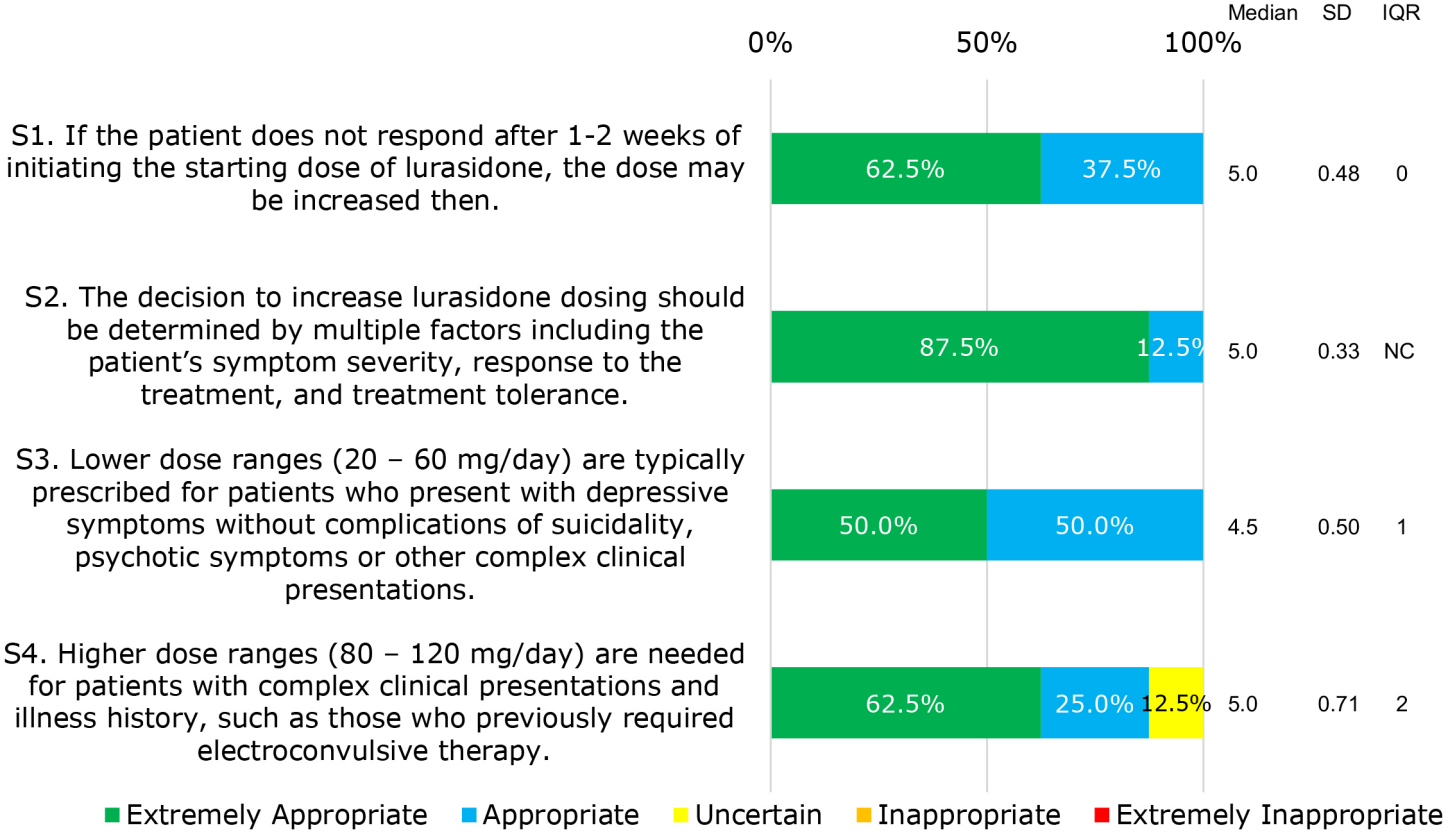
**Question 3: Considerations for increasing the lurasidone dose in adult bipolar I depression patients**. NC, not calculable.

Regarding increasing the lurasidone dose in adult patients [Fig. 3; Question 3 (Q3)], one voter was *uncertain* about using higher dose ranges [80–120 mg/day; Statement 4 (S4)] in adults with complex clinical presentations and illness histories.

For child/adolescent patients [**Supplementary Fig. 1**; Question 1B (Q1B)], two voters (25%) were *uncertain *if gastrointestinal upset occurred in susceptible patients given lurasidone 60 mg/day [Statement 5 (S5)], two voters were *uncertain *about reducing doses in children with comorbid psychiatric disorders requiring medication due to potential drug-drug interactions [Statement 9 (S9)], and three were uncertain about caution when prescribing lurasidone as children/adolescents may be more vulnerable to adverse effects [Statement 10 (S10)].

**Fig. 4.**
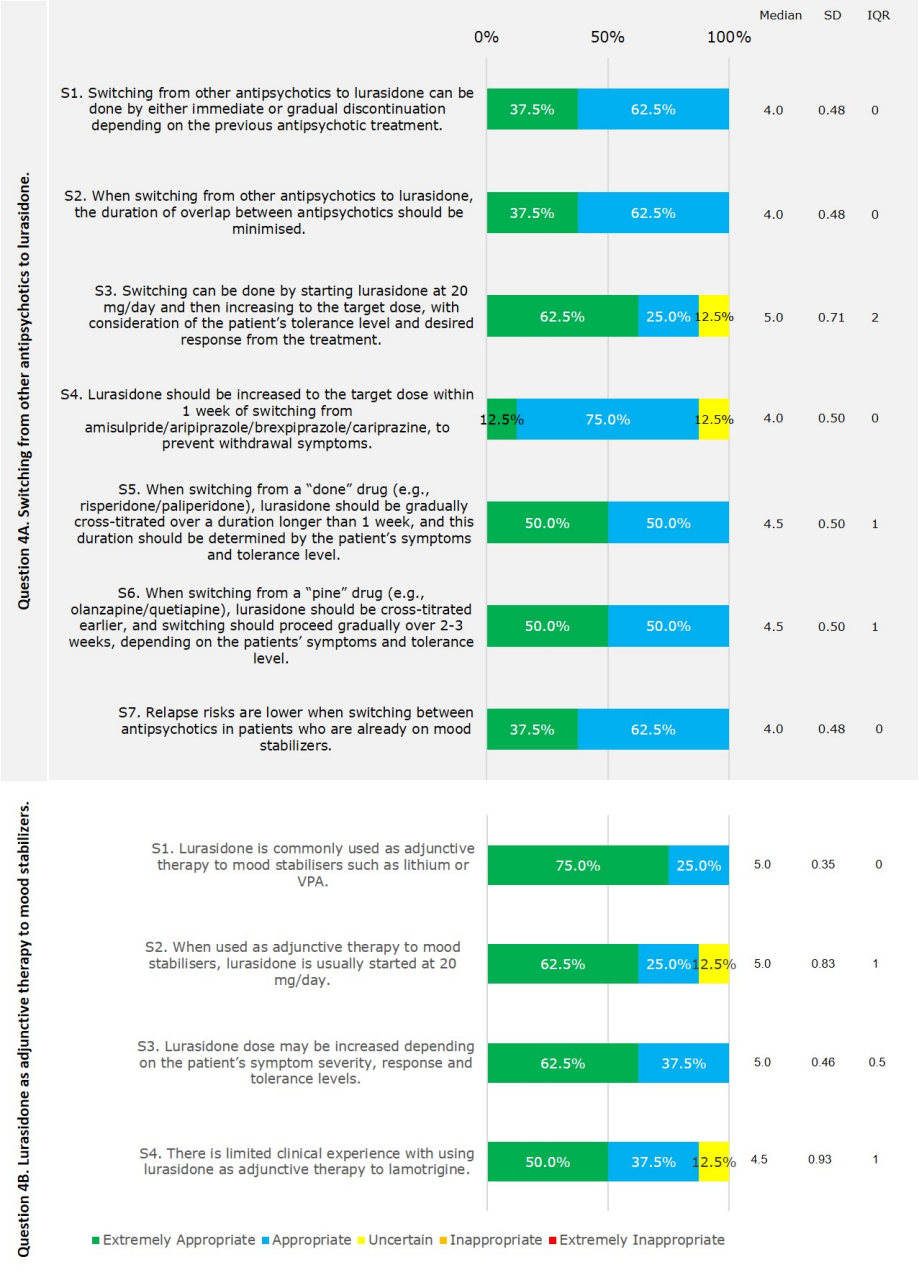
**Lurasidone: Switching or Adjunctive Therapy**. Question 4A: Switching from other antipsychotics to lurasidone. Question 4B: Lurasidone as adjunctive therapy to mood stabilizers. VPA, valproic acid.

**Fig. 5.**
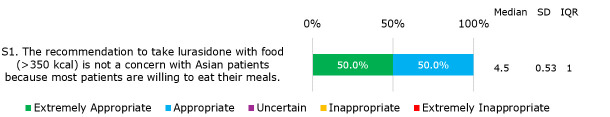
**Question 5: Importance of food intake**.

**Fig. 6.**
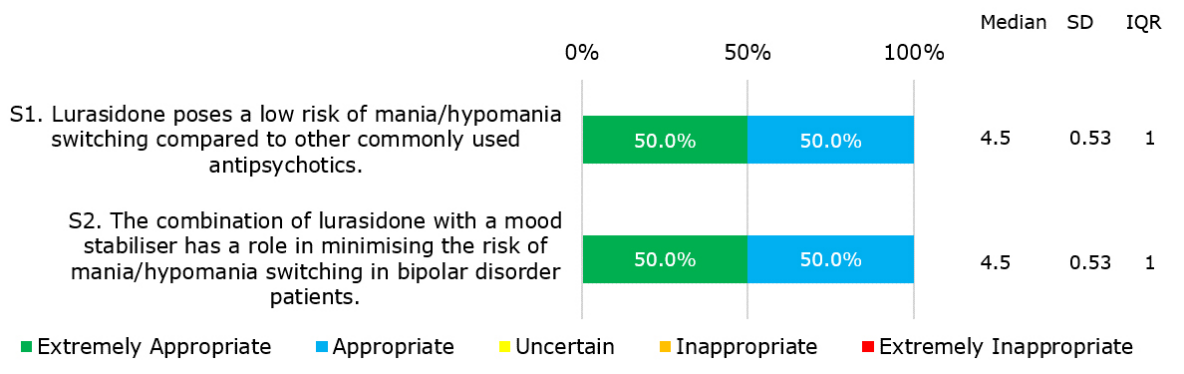
**Question 6: Risk of mania/hypomania switching**.

As for switching to lurasidone from other antipsychotics [Fig. 4; Question 4A (Q4A)], one voter was *uncertain* about switching by starting lurasidone at 20 mg/day and then increasing to the target dose with consideration of treatment tolerance levels and desired responses (S3). One voter was also *uncertain* about increasing lurasidone to the target dose within 1 week of switching from amisulpride/aripiprazole/brexpiprazole/cariprazine, to prevent withdrawal symptoms (S4). For the use of lurasidone as adjunctive therapy to mood stabilizers [Fig. 4; Question 4B (Q4B)], one voter was *uncertain *about starting lurasidone at 20 mg/day when used adjunctively to mood stabilizers (S2), and two voters were *uncertain* about there being limited clinical experience with using lurasidone as adjunctive therapy to lamotrigine (S4). The voters agreed that lurasidone should be taken with food since most Asian patients will eat their meals (Fig. 5; Question 5), that lurasidone posed a low risk of mania/hypomania switching (Fig. 6; Question 6), and that combining lurasidone with a mood stabilizer, including lamotrigine, minimized the risk of switching. However, there was limited clinical experience with combining lurasidone with lamotrigine [34].

This observation aligned with current evidence from randomized controlled trials that primarily evaluated lurasidone as adjunctive therapy with lithium or valproate [21,34]. A recent systematic review and meta-analysis confirmed that lurasidone had been studied in randomized controlled trials as adjunctive therapy to lithium or valproate monotherapy, but not in combination with lamotrigine [35]. Therefore, the panel’s consensus recommendations regarding lamotrigine combination therapy reflected both their clinical experience and alignment with the current evidence base.

**Fig. 7.**
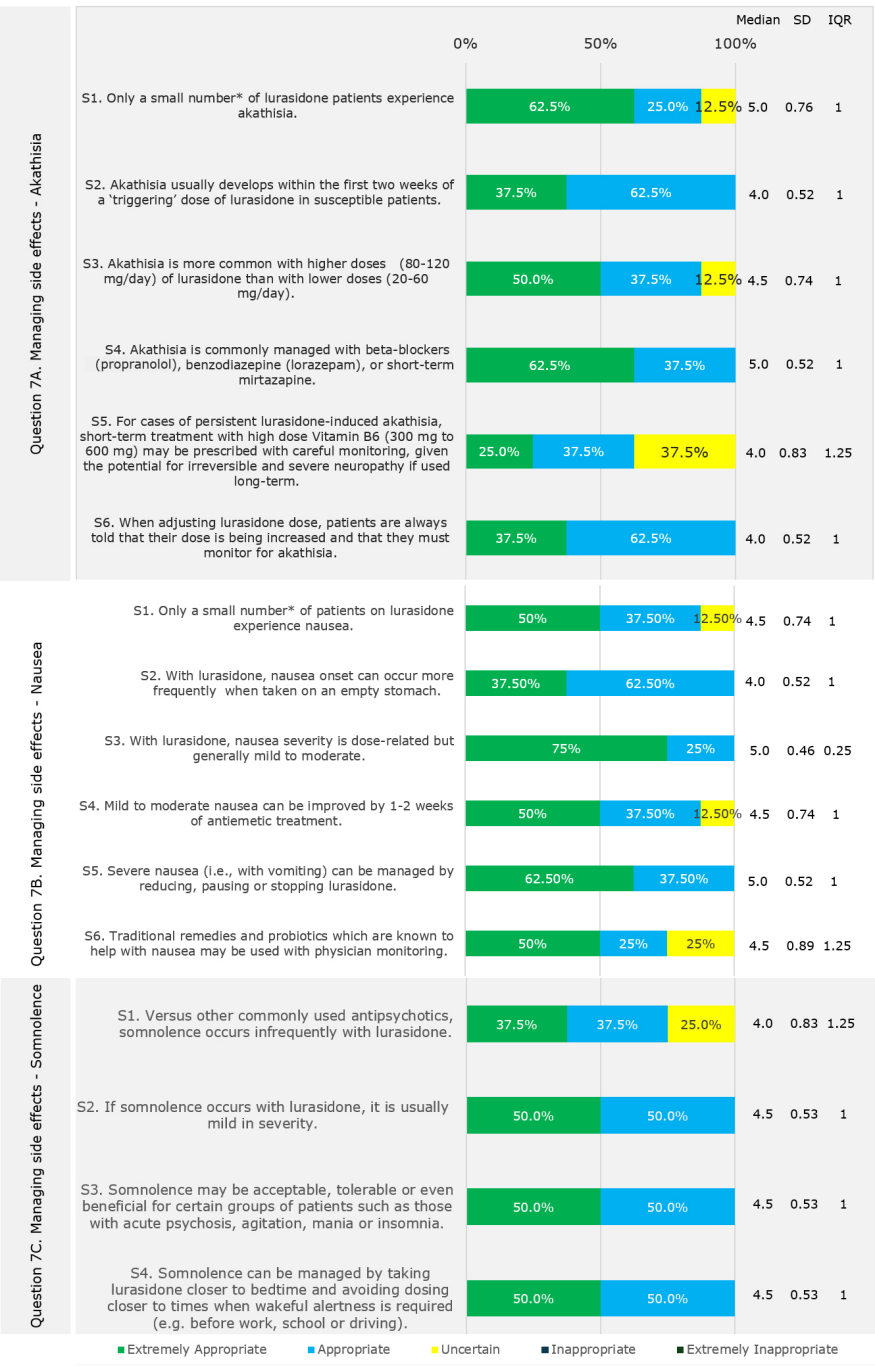
**Managing common side effects**. Question 7A: Akathisia. Question 7B: Nausea. Question 7C: Somnolence. *: Based on mean incidence in clinical trials [20,21,22].

On managing common side effects such as akathisia [Fig. 7; Question 7A (Q7A)], one voter was *uncertain *that only a small number of patients on lurasidone experienced it (S1), and one voter was *uncertain* about akathisia being more common at higher doses (80–120 mg/day) than at lower doses (20–60 mg/day) (S3). Three voters (37.5%) were *uncertain* about prescribing short-term, high-dose Vitamin B6 (300 mg to 600 mg), with careful monitoring, for patients with persistent lurasidone-induced akathisia (S5). As to managing common side effects such as nausea [Fig. 7 (Ref. [20,21,22]); Question 7B (Q7B)], one voter was *uncertain *(S1) that only a small number of patients on lurasidone experienced nausea, one voter was uncertain that mild-to-moderate nausea could be improved by 1–2 weeks of antiemetic treatment (S4), and two voters were *uncertain* about the use of traditional remedies and probiotics known to help with nausea (S6). On managing common side effects such as somnolence [Fig. 7; Question 7C (Q7C)], two voters were *uncertain *(S1) that somnolence occurred infrequently with lurasidone.

**Fig. 8.**
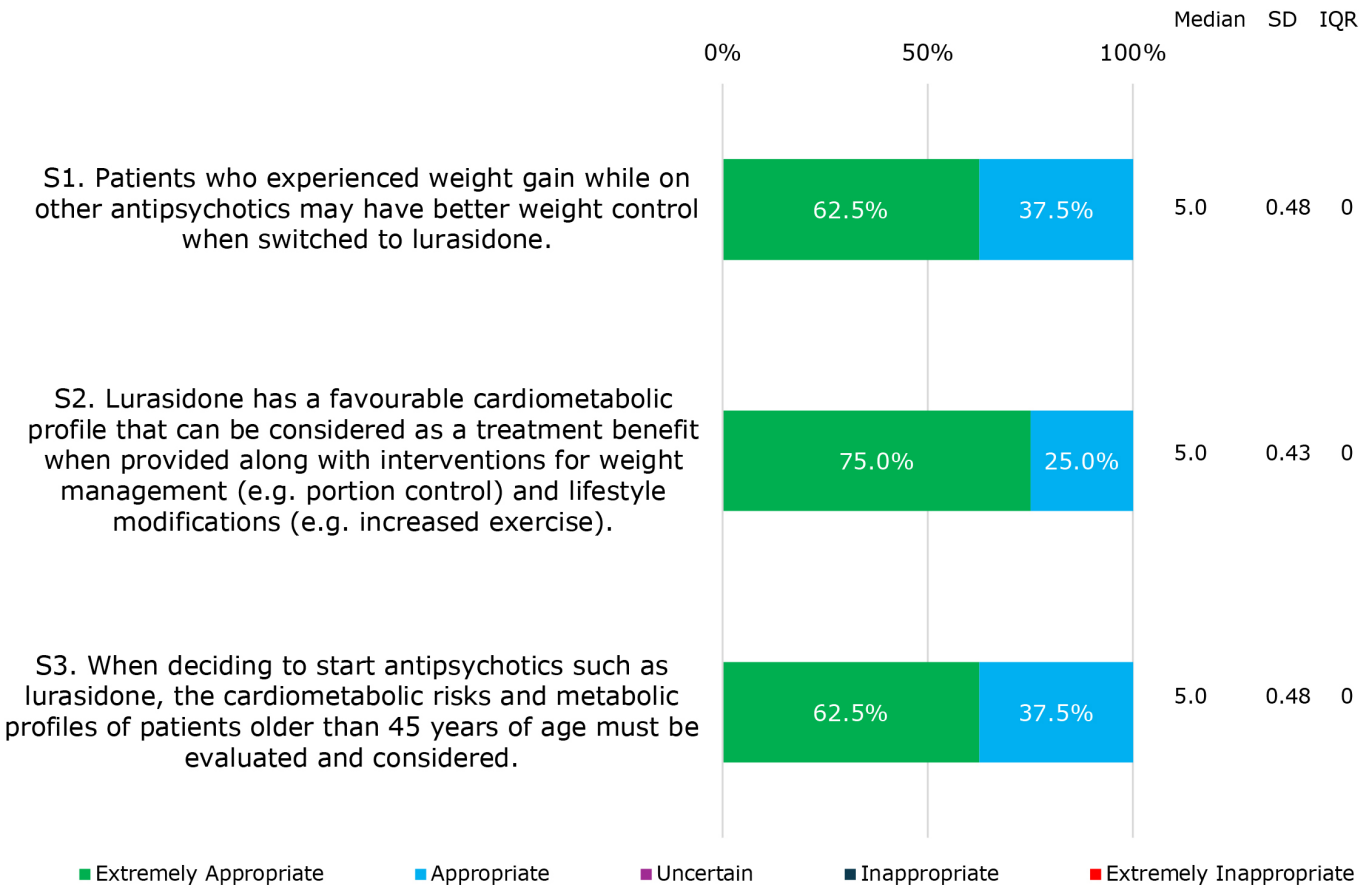
**Question 8: Lurasidone use in patients with cardiometabolic concerns**.

**Fig. 9.**
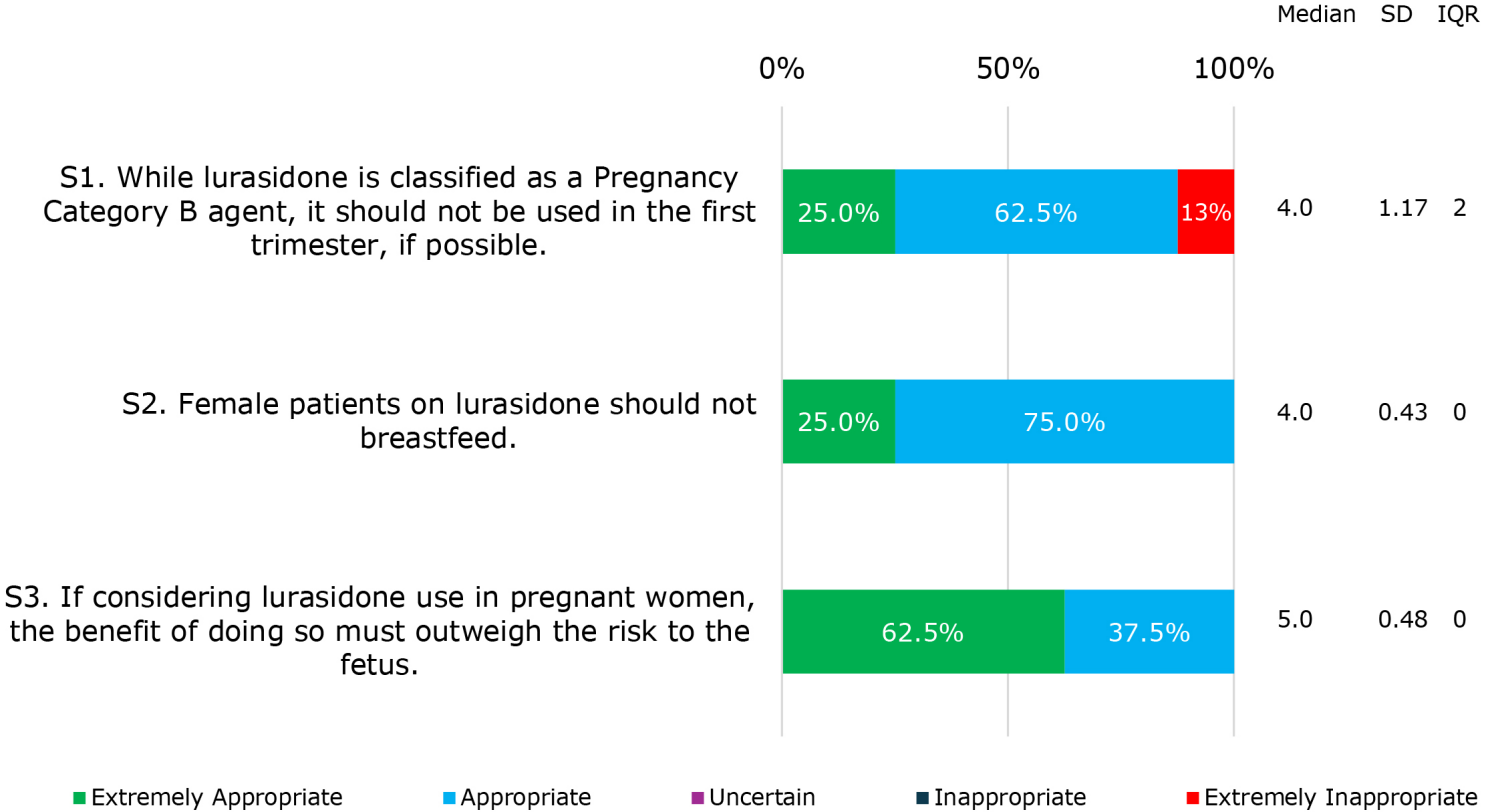
**Question 9: Lurasidone in female patients of child-bearing age**.

For special populations, the voters agreed with all statements on using lurasidone in patients with cardiometabolic concerns (Fig. 8). However, one voter considered it *extremely inappropriate* not to use lurasidone in the first trimester in female, child-bearing-age patients (Fig. 9; Question 9, S1).

## 4. Discussion

Most voters found nearly all recommendations for lurasidone in BD-I to be *appropriate* or *extremely appropriate*. It was *appropriate* for first-episode BD-I, especially in adult patients, to start at a dose of 20 mg/day and possibly increase it up to 120 mg/day. The voters also agreed that it is *appropriate* to consider multiple factors when increasing the lurasidone dose and to advise caution for elderly patients and those with renal/hepatic impairment. In addition, they agreed that it was *appropriate* to use lurasidone in child/adolescent patients at 20–80 mg/day, and that it had a low risk of mania/hypomania switching (relative to placebo, conventional antidepressants, or certain antipsychotics) [25,35], a favorable cardiometabolic profile, and generally manageable common side effects like akathisia, nausea, and somnolence. They agreed that lurasidone should be taken with food for optimal absorption and that it could be used as adjunctive therapy with mood stabilizers.

Statements voted as *uncertain*, *inappropriate,* and/or *extremely inappropriate* required additional discussion and clinical input. In nearly all cases, these votes reflected insufficient clinical encounters or insufficient experience with the specific cohort or scenario. For example, one voter questioned whether lurasidone could be titrated to 120 mg/day if 20 mg/day was ineffective (Fig. 2; Q2, S2), and another questioned whether most BD-I patients received 60–80 mg/day (Fig. 2; Q2, S3). We should note that lurasidone can have a linear dose-dependent effect [36] in bipolar depression, suggesting that higher doses improved depressive symptom severity more than did lower doses. However, a monotherapy study [22] found that higher lurasidone doses (80–120 mg/day) were no more effective than lower ranges (20–60 mg/day), perhaps because lurasidone-responsive patients likely received lower doses, whereas unresponsive patients had dose increases [36]. Regardless, most voters agreed that both statements were *appropriate* or *extremely appropriate*.

One *uncertain *vote was given to the statement that elderly BD-I patients had a usual dose range of 20–80 mg/day (**Supplementary Fig. 1**; Q1A, S1) because the lurasidone clinical trial program for this indication had few such patients (**Supplementary Fig. 1**; Q1A, S4). Although there was a general lack of lurasidone safety and efficacy data for elderly patients, those votes were likely due to insufficient clinical encounters or experience with the cohort or situation. Regardless, in one post hoc evaluation for bipolar depression in patients ≥55 years [37], monotherapy lurasidone (20–60 mg/day or 80–120 mg/day) led to a significantly greater mean change in MADRS score than did placebo (−14.8 vs −7.1; *p* = 0.003), although only 11% of patients were ≥65 years. Caution is advised when prescribing lurasidone to this group due to their higher rates of comorbidities [37] and because of age-related changes in albumin and alpha-1 acid glycoprotein levels, both of which are proteins that bind to lurasidone and can affect its pharmacokinetics.

Gastrointestinal upset was a common adverse effect in children and adolescents, often linked to individual vulnerability; some susceptible patients may have experienced this issue even at low lurasidone doses (e.g., 20 mg/day). Our expert clinical consensus advised caution for gastrointestinal upset at a daily treatment dosage of around 60 mg. In treating child and adolescent patients, the guiding principle is to start with a low dose and increase slowly, with frequent clinical monitoring for both response and adverse effects. Dosage is contingent on the clinical scenario, which is typically heterogeneous and changeable. To avoid harmful drug-drug interactions, lurasidone dosage should be carefully assessed in conjunction with the patient’s other medications. Therefore, as our consensus indicated, dose adjustments should be based on the patient’s treatment response and clinical condition. Lurasidone is indicated for children and adolescents aged 10–18 years, a period that includes many developmental changes. Some experts may consider adolescents nearing 18 years as adults, with minimal differences in lurasidone responses and adverse effects.

Our voters, including one child/adolescent psychiatrist, agreed on most statements for lurasidone usage in child/adolescent BD-I patients. *Uncertain *votes reflected limited clinical encounters with this cohort. These statements were that gastrointestinal upset may occur in susceptible child/adolescent patients (**Supplementary Fig. 1**; Q1B, S5) at lurasidone 60 mg/day; that children/adolescents with comorbid psychiatric disorders requiring medication due to potential drug-drug interactions may need lurasidone dose reduction (**Supplementary Fig. 1**; Q1B, S9); and that caution is required when prescribing lurasidone, as this cohort may be vulnerable to adverse effects. Children/adolescents with BD-I who received at least 1 dose of monotherapy lurasidone (mean dose 33.6 mg/day; dose range: 20–80 mg/day) had statistically signiﬁcant improvements in depression, anxiety, quality of life, and general functioning than they did with placebo treatment [38]. However, children/adolescents reported higher incidences of nausea, vomiting, and upper abdominal pain than did adult patients on lurasidone monotherapy or placebo [39,40]. Children/adolescents with comorbid psychiatric disorders (e.g., schizophrenia, attention-deficit/hyperactivity disorder) requiring medications with similar mechanisms may require lurasidone dose reductions to minimize drug-drug interaction risks that lead to overdose or adverse neuromotor and/or metabolic effects [41]. One study noted that 80.63% of children/adolescents on antipsychotic polypharmacy received at least two SGAs, whereas 19.37% received a combined first-generation antipsychotic (FGA) with an SGA [41]. SGAs, such as lurasidone, have minimal or no extrapyramidal symptoms and are considered safer than FGAs, but care is needed with their concomitant use as they can lead to, or increase the risk of, serious adverse events such as hyperlipidemia and type II diabetes. Moreover, younger patients with complex psychiatric disorders may also require lower lurasidone doses to avoid exacerbating the existing disorders [42]. In child/adolescent patients with schizophrenia or bipolar disorder, cognitive dysfunction is common, and lurasidone shows treatment benefits in several cognitive domains, most notably in visual learning and processing speed [38].Non-psychotic symptoms, especially cognitive function impairment, are closely associated with prognosis. Thus, in child/adolescent patients, lurasidone treatment plays an important role in cognitive function and lifelong function.

The statement on switching antipsychotics by starting lurasidone at 20 mg/day and then increasing it to the target dose, with consideration of the patient’s tolerance level and desired response from the treatment (Fig. 4; Q4A, S3), received one *uncertain *vote due to insufficient clinical encounters or experiences with switching by starting at 20 mg/day. Similarly, the statement that lurasidone should be increased to the target dose within 1 week of switching from amisulpride, aripiprazole, brexpiprazole, or cariprazine, to prevent withdrawal symptoms (Fig. 4; Q4A, S4) also received one *uncertain *vote. Withdrawal symptoms, including somatic (e.g., nausea, sweating), motor, and psychological (e.g., psychosis) effects, can occur when stopping both FGA and SGA [43,44,45] and when stopping antipsychotic medication abruptly. Furthermore, lurasidone only reaches effective steady-state plasma levels in seven days, and some of these medications (e.g., amisulpride) may have washed out before that occurs.

The statement that lurasidone is usually started at 20 mg/day as adjunctive therapy to mood stabilizers (Fig. 4; Q4B, S2) received one *uncertain* vote. The lowest dose of lurasidone formulated is 20 mg; therefore, starting at 20 mg/day is reasonable [22,23,36,39], including in patients taking moderate Cytochrome P450 family 3 subfamily A member 4 inhibitors or with severe renal impairment. There are few efficacy and safety studies on adjunctive therapy in bipolar depression, and their outcomes differ [21,23]. However, one systematic review and meta-analysis [34] reported both greater benefits and risks in patients with bipolar depression on adjunctive SGAs such as lurasidone to lithium or valproate, without significant differences in severe adverse events. Although decision-making should be shared and the patient’s clinical status and environment should be considered when providing adjunctive therapy, monotherapy lurasidone used adjunctively with lithium or valproate is a recommended first-line treatment for bipolar depression in several guidelines [26,30,34,46]. Two voters felt that there were insufficient clinical encounters or experiences with lurasidone adjunctive therapy, resulting in their being *uncertain* about their clinical experience levels with lurasidone adjunctive therapy (Fig. 4; Q4B, S4).

In an adult, short-term, placebo-controlled, premarketing study [47,48], monotherapy lurasidone was compared at lower doses (20–60 mg/day) and higher doses (80–120 mg/day) with placebo in patients with bipolar depression (*n *= 331). The most common dose-related (up to 111 mg/day) side effects (≥5% and at least twice the rate of placebo) were extrapyramidal symptoms (4.9% vs 9.0%, respectively), somnolence (7.3% vs 13.8%, respectively), akathisia (7.9% vs 10.8%, respectively), and nausea (10.4% vs 17.4%, respectively) [48].

Three statements on managing akathisia received *uncertain* votes, including: only a small number of lurasidone patients experienced akathisia (Fig. 7; Q7A, S1), which aligned with mean incidence rates of 8–14% in trials [21,22,24]. This was most likely due to a lack of clinical encounters or experience with akathisia among patients treated by the voter. Also, although the statement (Fig. 7; Q7A, S3) on akathisia being more common with higher (80–120 mg/day) than with lower lurasidone doses (20–60 mg/day) received one *inappropriate *vote, nearly all voters agreed that akathisia is more common with higher doses, as shown in the premarketing study [47,48]. Again, nearly all voters agreed that short-term, high-dose Vitamin B6 (300 mg to 600 mg) may be prescribed for persistent lurasidone-induced akathisia (Fig. 7; Q7A, S5) with careful monitoring given the potential for irreversible and severe neuropathy if used long-term. As recently reported [49], Vitamin B6 may improve akathisia [50] but is a relatively new intervention for lurasidone-associated akathisia. This newness and lack of experience with Vitamin B6 in this context were the likely reasons for an *uncertain *vote. Lack of experience is also a likely reason for the *uncertain* vote on akathisia occurring at either dose range. One *uncertain *vote was given to the statements that only a small number of patients on lurasidone experience nausea (Fig. 7; Q7B, S1) (which aligned with mean incidence rates of 8%–14% in trials [21,22,24]), that nausea can occur more frequently when lurasidone was taken on an empty stomach (Fig. 7; Q7B, S2), and that mild-to-moderate nausea could be improved by 1–2 weeks of antiemetic treatment (Fig. 7; Q7B, S4). Again, these responses were likely due to a lack of clinical encounters or experience with lurasidone-associated nausea. Lurasidone exhibits partial agonism of 5-HT1A receptors, which may cause nausea at doses lower than 160 mg/day [51]. Notably, experienced European psychiatrists providing guidance on lurasidone-associated side effects [52] agreed that nausea is infrequent with lurasidone, perhaps because it needs to be taken with food. Regardless, antiemetics are typically prescribed in routine clinical practice for nausea, whether induced by lurasidone or not. Similarly, except for one *uncertain* vote due to a lack of clinical experience with the situation, the voters agreed with physician monitoring when using traditional remedies and probiotics known to ease nausea (Fig. 7; Q7B, S6). The statement that somnolence occurred infrequently with lurasidone received two *uncertain* votes (Fig. 7; Q7C, S1) and aligned with mean incidence rates of 13.6–17% in trials [21,22,24]. Although many antipsychotic medications are linked to somnolence, lurasidone is less sedating than other atypical antipsychotics (e.g., quetiapine, olanzapine, risperidone) [53], even at elevated doses. Patients switching to lurasidone from a sedating antipsychotic may benefit from prolonging cross-titration periods to reduce the risk of rebound effects such as insomnia, whereas those newly initiating lurasidone may experience less drowsiness with evening doses.

Our statement on not using lurasidone during the first trimester, where possible, resulted in one *extremely inappropriate *vote (Fig. 9; Q9, S1), again likely due to a lack of clinical encounter or experience with such patients. It may have also stemmed from the conviction that lurasidone should be used in the first trimester, even though our cautious statement specified the use of lurasidone if possible. Untreated bipolar disorder during pregnancy is associated with poor prenatal care, decreased fetal growth, and increased risks of postpartum complications, including psychosis, adverse neurodevelopmental outcomes, suicide and filicide [54,55], and higher postpartum relapse rates [56]. However, concerns that typical antipsychotic drug usage in the third trimester was linked to extrapyramidal and withdrawal symptoms in neonates [57], resulted in the more frequent use of atypical antipsychotics [58], such as lurasidone. Although replaced by the United States Food and Drug Administration’s Pregnancy and Lactation Labeling Rule (PLLR) in 2016 [58], lurasidone’s Pregnancy Category B classification remains widely recognized in Asia-Pacific psychiatric practice. Clinicians should consider current PLLR standards [59] and international guidelines [58] alongside Category B when assessing pregnancy risks. Lurasidone use requires clear clinical benefits that outweigh fetal risks [58], with shared decision-making and obstetric consultation [54,60]. Lurasidone should be used cautiously in pregnancy, especially in the first trimester when organogenesis occurs. However, it may be considered in specific situations: severe BD-I depression unresponsive to safer first-line mood stabilizers; documented failures with standard antimanic agents; or a high relapse risk where maternal benefit outweighs potential fetal risks. Limited perinatal data exist regarding lurasidone exposure during pregnancy [60]; therefore, treatment decisions should be highly individualized and made collaboratively with both the patient and obstetric colleagues using shared decision-making frameworks [54,60].

### Limitations

This consensus study used rigorous RAND/UCLA methods to generate expert recommendations from an eight-member panel representing five Asian regions/territories where lurasidone is approved and marketed. The panel size fell within the validated RAND/UCLA range (typically 7–13) [32] and aligned with comparable regional initiatives [27], but the geographic scope did not encompass all Asian regions (e.g., South Asia, West Asia, Central Asia). Clinicians outside these regions should consider local pharmacotherapy availability, treatment guidelines, patient demographics, and cultural factors when applying these recommendations.

Additional methodological limitations included the absence of a comprehensive systematic review, which could have minimized evidence-selection bias and enhanced reproducibility. Recommendations for special populations (elderly patients, pregnant women) may have relied on inferences from less-specific data due to current evidence gaps. In addition, the multinational scope precluded specific guidance on cost, accessibility, or implementation [27,61].

## 5. Conclusions

This expert consensus provided practical guidance on using lurasidone for BD-I in Asian patients, based on clinical experiences from psychiatrists across several Asian countries. Key recommendations included using lurasidone as a first-line treatment for first-episode BD-I in adults, starting at 20 mg/day and titrating up to 120 mg/day if needed, with most patients receiving 60–80 mg/day. For elderly patients, a lower dose range of 20–80 mg/day was recommended, and children/adolescents can have 20–80 mg/day with careful monitoring. Lurasidone has a low risk of mania/hypomania switching, a favorable cardiometabolic profile, and generally manageable, common side effects. It should be taken with food and can be used as an adjunctive therapy with mood stabilizers. Caution was advised for pregnant women, especially during the first trimester. This consensus thus provided a valuable Asian perspective to complement the existing guidelines on lurasidone use in bipolar depression.

## Data Availability

Data is available from the corresponding author upon reasonable request.

## Supplementary Material

Supplementary material associated with this article can be found, in the online version, at https://doi.org/10.31083/AP49358.
