# Supplementary material for: Expert Consensus on the Use of Lurasidone for Bipolar I Depression in Asian Patients
Source: Alpha Psychiatry. 2026 Jun 30;27(3):49358. doi: 10.31083/AP49358 (PMC13339877; doi:10.31083/AP49358)
Supplement: Supplementary file 1 [file 2757-8038-27-3-49358-s1.zip › Supplementary Material.docx]

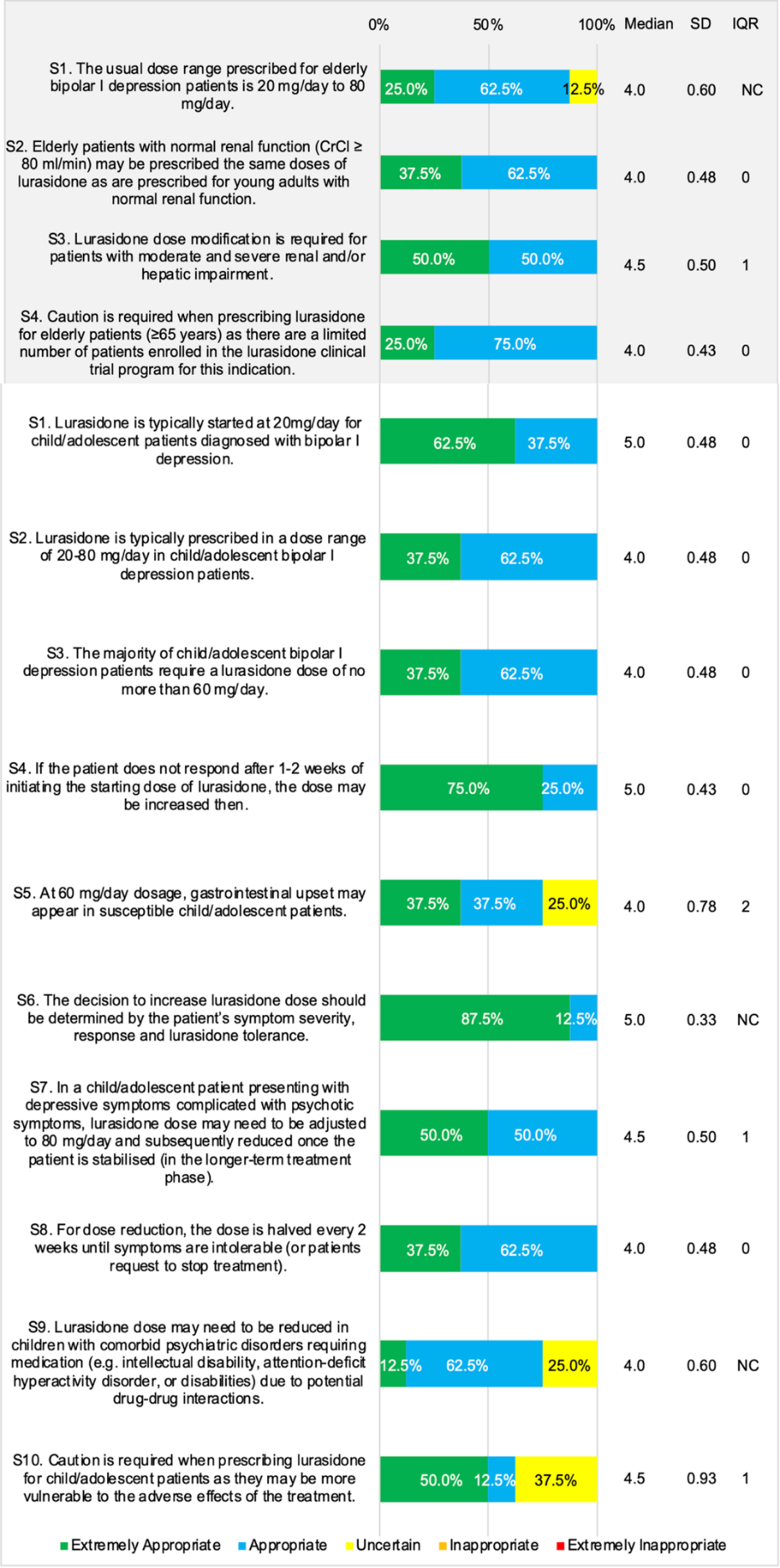


Question 1A. Considerations for using lurasidone in elderly bipolar I depression patients (≥65 years of age).

Question 1B. Lurasidone usage in child/adolescent bipolar I depression patients.

**Supplementary Fig. 1. Question 1A. Considerations for using lurasidone in elderly bipolar I depression patients (≥65 years of age). Question 1B. Lurasidone usage in child/adolescent bipolar I depression patients.** CrCl, Creatinine Clearance; SD, Standard Deviation; IQR, Interquartile Range; NC, Not Calculable.

Supplementary Table 1. All Statements Reaching Agreement and Therefore Consensus.

| Category | Statements with agreement leading to consensus |
| --- | --- |
| Question 1. Lurasidone for patients who present with first-episode bipolar I depression. | (1) S1: Lurasidone is suitable for adult patients who present with a first bipolar I depressive episode.  (2) S2: Lurasidone is suitable for child/adolescent patients who present with a first episode bipolar I depression. |
| Question 2. Lurasidone usage in adult bipolar I depression patients. | (1) S1: Lurasidone is typically started at 20mg/day for adult patients diagnosed with bipolar I depression.  (2) S2: If the desired/expected response is not achieved with the starting dose of lurasidone 20 mg/day, the dose can be increased up to a maximum of 120 mg/day.  (3) S3: Most bipolar I depression patients are prescribed a lurasidone dose of 60–80mg/day. |
| Question 3. Considerations for increasing lurasidone dose in adult bipolar I depression patients. | (1) S1: If the patient does not respond after 1–2 weeks of initiating the starting dose of lurasidone, the dose may be increased then.  (2) S2: The decision to increase lurasidone dosing should be determined by multiple factors including the patient’s symptom severity, response to the treatment, and treatment tolerance.  (3) S3: Lower dose ranges (20–60 mg/day) are typically prescribed for patients who present with depressive symptoms without complications of suicidality, psychotic symptoms or other complex clinical presentations.  (4) S4: Higher dose ranges (80–120 mg/day) are needed for patients with complex clinical presentations and illness history, such as those who previously required electroconvulsive therapy. |
| Supplementary Fig. 1, Question 1A. Considerations for using lurasidone in elderly bipolar I depression patients (≥65 years of age). | (1) S1: The usual dose range prescribed for elderly bipolar I depression patients is 20–80 mg/day  (2) S2: Elderly patients with normal renal function (CrCl ≥80 mL/min) may be prescribed the same doses of lurasidone as are prescribed for young adults with normal renal function.  (3) S3: Lurasidone dose modification is required for patients with moderate and severe renal and/or hepatic impairment.  (4) S4: Caution is required when prescribing lurasidone for elderly patients (≥65 years) as there are a limited number of patients enrolled in the lurasidone clinical trial program for this indication. |
| Supplementary Fig. 1, Question 1B. Lurasidone usage in child/adolescent bipolar I depression patients. | (1) S1: Lurasidone is typically started at 20 mg/day for child/adolescent patients diagnosed with bipolar I depression.  (2) S2: Lurasidone is typically prescribed in a dose range of 20–80 mg/day in child/adolescent bipolar I depression patients.  (3) S3: The majority of child/adolescent bipolar I depression patients require a lurasidone dose of no more than 60 mg/day.  (4) S4: If the patient does not respond after 1–2 weeks of initiating the starting dose of lurasidone, the dose may be increased then.  (5) S5: At 60 mg/day dosage, gastrointestinal upset may appear in susceptible child/adolescent patients.  (6) S6: The decision to increase lurasidone dose should be determined by the patient’s symptom severity, response and lurasidone tolerance.  (7) S7: In a child/adolescent patient presenting with depressive symptoms complicated with psychotic symptoms, lurasidone dose may need to be adjusted to 80 mg/day and subsequently reduced once the patient is stabilized (i.e., in the longer-term treatment phase).  (8) S8: For dose reduction, the dose is halved every 2 weeks until symptoms are intolerable (or patients request to stop treatment).  (9) S9: Lurasidone dose may need to be reduced in children with comorbid psychiatric disorders requiring medication (e.g., intellectual disability, attention-deficit hyperactivity disorder, or disabilities) due to potential drug-drug interactions.  (10) S10: Caution is required when prescribing lurasidone for child/adolescent patients as they may be more vulnerable to the adverse effects of the treatment. |
| Question 4A. Switching to lurasidone from other antipsychotics. | (1) S1: Switching from other antipsychotics to lurasidone can be done by either immediate or gradual discontinuation depending on the previous antipsychotic treatment.  (2) S2: When switching from other antipsychotics to lurasidone, the duration of overlap between antipsychotics should be minimized.  (3) S3: Switching can be done by starting lurasidone at 20 mg/day and then increasing to the target dose, with consideration of the patient’s tolerance level and desired response from the treatment.  (4) S4: Lurasidone should be increased to the target dose within 1 week of switching from amisulpride/aripiprazole/brexpiprazole/cariprazine, to prevent withdrawal symptoms.  (5) S5: When switching from a “done” drug (e.g., risperidone/paliperidone), lurasidone should be gradually cross-titrated over a duration longer than 1 week, and this duration should be determined by the patient’s symptoms and tolerance level.  (6) S6: When switching from a “pine” drug (e.g., olanzapine/quetiapine), lurasidone should be cross-titrated earlier, and switching should proceed gradually over 2–3 weeks, depending on the patients’ symptoms and tolerance level.  (7) S7: Relapse risks are lower when switching between antipsychotics in patients who are already on mood stabilizers. |
| Question 4B. Lurasidone as adjunctive therapy to mood stabilizers. | (1) S1: Lurasidone is commonly used as adjunctive therapy to mood stabilizers such as lithium or valproate.  (2) S2: When used as adjunctive therapy to mood stabilizers, lurasidone is usually started at 20 mg/day.  (3) S3: Lurasidone dose may be increased depending on the patient’s symptom severity, response and tolerance levels.  (4) S4: There is limited clinical experience with using lurasidone as adjunctive therapy to lamotrigine. |
| Question 5. Importance of food intake. | (1) S1: The recommendation to take lurasidone with food (>350 kcal) is not a concern with Asian patients because most patients are willing to eat their meals. |
|  |  |
| Question 6: Risk of mania/hypomania switching. | (1) S1: Lurasidone poses a low risk of mania/hypomania switching.  (2) S2: The combination of lurasidone with a mood stabilizer, such as lamotrigine, has a role in minimizing the risk of mania/hypomania switching in bipolar disorder patients. |
| Question 7A. Managing common side effects - Akathisia. | (1) S1: Only a small number* of lurasidone patients experience akathisia.  (2) S2: Akathisia usually develops within the first two weeks of a ‘triggering’ dose of lurasidone in susceptible patients.  (3) S3: Akathisia is more common with higher doses (80–120 mg/day) of lurasidone than with lower doses (20–60 mg/day).  (4) S4: Akathisia is commonly managed with beta-blockers (propranolol), benzodiazepine (lorazepam), or short-term mirtazapine.  (5) S5: For cases of persistent lurasidone-induced akathisia, short-term treatment with high-dose Vitamin B6 (300–600 mg) may be prescribed with careful monitoring, given the potential for irreversible and severe neuropathy if used long-term.  (6) S6: When adjusting lurasidone dose, patients are always told that their dose is being increased and that they must monitor for akathisia. |
| Question 7B. Managing common side effects – Nausea. | (1) S1: Only a small number* of patients on lurasidone experience nausea.  (2) S2: With lurasidone, nausea onset can occur more frequently when taken on an empty stomach.  (3) S3: With lurasidone, nausea severity is dose-related but generally mild to moderate.  (4) S4: Mild-to-moderate nausea can be improved by 1–2 weeks of antiemetic treatment.  (5) S5: Severe nausea (i.e., with vomiting) can be managed by reducing, pausing or stopping lurasidone.  (6) S6: Traditional remedies and probiotics which are known to help with nausea may be used with physician monitoring. |
| Question 7C. Managing common side effects – Somnolence. | (1) S1: Versus other commonly used antipsychotics, somnolence occurs infrequently with lurasidone.  (2) S2: If somnolence occurs with lurasidone, it is usually mild in severity.  (3) S3: Somnolence may be acceptable, tolerable or even beneficial for certain groups of patients such as those with acute psychosis, agitation, mania or insomnia.  (4) S4: Somnolence can be managed by taking lurasidone closer to bedtime and avoiding dosing closer to times when wakeful alertness is required (e.g., before work, school or driving). |
| Question 8. Lurasidone in patients with cardiometabolic concerns. | (1) S1: Patients who experienced weight gain while on other antipsychotics may have better weight control when switched to lurasidone.  (2) S2: Lurasidone has a favorable cardiometabolic profile that can be considered as a treatment benefit when provided along with interventions for weight management (e.g., portion control) and lifestyle modifications (e.g., increased exercise).  (3) S3: When deciding to start antipsychotics such as lurasidone, the cardiometabolic risks and metabolic profiles of patients older than 45 years must be evaluated and considered. |
| Question 9. Lurasidone in female child-bearing age patients. | (1) S1: While lurasidone is classified as a Pregnancy Category B agent, it should not be used in the first trimester, if possible.  (2) S2: Female patients on lurasidone should not breastfeed.  (3) S3: If considering lurasidone use in pregnant women, the benefit of doing so must outweigh the risk to the fetus. |
